# Bacterial species-specific modulatory effects on phenotype and function of camel blood leukocytes

**DOI:** 10.1186/s12917-021-02939-1

**Published:** 2021-07-12

**Authors:** Jamal Hussen

**Affiliations:** Department of Microbiology, College of Veterinary Medicine, King Faisal University, Al-Ahsa, Saudi Arabia

**Keywords:** Bacterial stimulation, Monocyte subsets, Granulocytes, Flow cytometry, Dromedary camel

## Abstract

**Background:**

Recent studies have reported pathogen-species-specific modulating effects on the innate immune system. *Escherichia coli*, *Staphylococcus aureus*, and *Streptococcus agalactiae* are important pathogenic bacteria responsible for different infectious diseases in several animal species. In the present study, a whole blood culture with S. *aureus*, E. *coli*, or S. *agalactiae* and flow cytometry were used to investigate, whether stimulation with different bacterial species induces different immunomodulation patterns in camel leukocytes. The expression of different cell surface myeloid markers and cell adhesion molecules on monocytes and neutrophils was investigated. In addition, the capacity of monocytes and neutrophils to produce reactive oxygen species (ROS) was analyzed.

**Results:**

Stimulation with either of the bacterial species resulted in the expansion of the camel CD14^high^MHCII^high^ monocyte subset with a reduced fraction of CD14^high^MHCII^low^ monocytes. For the CD14^low^MHCII^high^ monocytes, however, only stimulation with S. *aureus* or S. *agalactiae* increased their fractions in blood. Although all bacterial species elicited the upregulation of cell surface MHC class II molecules on granulocytes, the increase was, however, highest on cells stimulated with *S. aureus*. The expression levels of the two adhesion molecules, CD11a and CD18, on neutrophils and monocytes were differently affected by bacterial stimulation. Functionally, *E. coli* failed to stimulate ROS production in monocytes, while induced a strong ROS production response in granulocytes. S. *agalactiae* elicited a week ROS production in granulocytes when compared to the other two pathogens.

**Conclusions:**

The different responsiveness of monocytes and granulocytes toward different bacterial species indicates different host-pathogen interaction mechanisms for the two cell populations. In addition, the phenotypic and functional differences between cells stimulated with E. *coli*, S. *aureus*, or S. *agalactiae* suggests pathogen-species-specific modulating effects of the bacterial pathogens on the camel innate myeloid cells.

## Background

 Pathogen-species-specific modulatory effects on the innate immune system have been described in several species and for different pathogens [1–9]. *Escherichia coli (E. coli)*, *Staphylococcus aureus* (*S*. *aureus*), and *Streptococcus agalactiae* (*S*. *agalactiae*) are major causative agents of different infectious diseases in the dromedary camel including metritis, mastitis, and neonatal infections [2, 10]. For the three different bacterial species, different host-pathogen interaction mechanisms and different disease outcomes have been reported [11, 12]. In animals with mastitis, infection with *E. coli* results mostly in severe acute inflammatory disease with clinical signs [1, 13], whereas *S. aureus* and *S. agalactiae* are mainly responsible for mild subclinical infections of the udder [1, 3, 5, 14]. Furthermore, gram-negative (*E. coli* ) and gram-positive bacteria (*S. aureus* and *S*. *agalactiae*) differ in their pathogenesis mechanisms. While gram-negative bacteria release the endotoxin lipopolysaccharide (LPS) [13, 15], gram-positive bacteria rely on exotoxins as virulence factors [15, 16]. In a recent study, the systemic effects of local challenging of the bovine mammary gland with *S. aureus* or *E. coli* on some innate immune functions of the liver have been comparatively investigated [8]. Both bacterial species affected the liver transcriptome with pathogen-specific strategies to modulate the innate immune response. While *E. coli* significantly downregulated key components of the complement system, *S. aureus* inhibited the cell signaling via integrin, FcγR and Rho GTPases in the liver [8]. In a mouse model for *S. aureus* and *E. coli* infections, animals with *E. coli* infection showed significantly greater serum levels of the cytokines interleukin (IL)-1α, IL-1β, IL-6, monocyte chemotactic protein (MCP)-1, and macrophage inflammatory protein (MIP)-1α than *S. aureus-*infected mice [17].

Monocytes and neutrophils are innate cell populations with key roles during the immune response to bacterial infections [18–20]. The significant roles of camel monocytes and neutrophils in the pathophysiologic response to bacterial pathogens have been described in some recent studies. Camel bacterial endometritis was characterized by leukocytosis with significant neutrophilia [21, 22]. In addition, bacterial clinical endometritis in female camels resulted in a significant expansion of camel inflammatory monocytes. The rise in camel inflammatory monocytes has been also found indicative of endometritis severity grade [22]. Furthermore, in vitro stimulation of camel neutrophils with lipopolysaccharide (LPS), the pathogen-associated molecular pattern from E. coli, induced a significant impairment of their phagocytosis, while their ROS generating potential remained unchanged [23].

Based on the surface expression of the lipopolysaccharide (LPS) co-receptor CD14 and the major histocompatibility complex (MHC) class II molecules, camel monocytes have been recently classified into monocyte subset Mo-I (CD14^high^MHCII^low^), monocyte subset Mo-II (CD14^high^MHCII^high^), and monocyte subset Mo-III (CD14^low^MHCII^high^) [24]. Several phenotypic and functional differences were identified between the three subsets of camel monocytes [24].

In addition to their essential role as first responders during the innate immune response [18, 19], increasing evidence also suggests that neutrophilic granulocytes may contribute to adaptive immunity by processing and presenting antigens to T cells [25]. Upon antigen-specific stimulation, human neutrophilic granulocytes acquire MHC class II molecules and different co-stimulatory molecules rendering them to antigen-presenting cells able to stimulate CD4-positive T cell responses [26].

Studies on the pathogen-species-specific effect of bacterial pathogens on the innate immune system of camels are limited. The aim of the present study was, therefore, to analyze changes in phenotype and function of camel leukocytes in a whole blood culture with different bacterial species.

## Materials and methods

### Animals and blood collection

 Blood samples were collected from six apparently healthy camels housed at the Camel Research Center, King Faisal University, Al-Ahsa, Saudi Arabia. The studied animals included male (to exclude the effect of reproductive physiology like pregnancy or parturition) dromedary camels (*Camelus dromedarius*) aged between 8 and 10 years with comparable body condition scores. All animals were fed on hay and barley in addition to a mineral supplement. Water was available ad libitum. Blood was obtained by venipuncture of the external jugular vein (*vena jugularis externa*) into vacutainer tubes containing EDTA (Becton Dickinson, Heidelberg, Germany). The animals were only used for blood collection. Collected blood samples were stimulated ex vivo.

### Whole blood stimulation with live bacteria

Whole blood stimulation was performed according to a previously established method [27]. The bacterial species used for in vitro stimulation included S. *aureus*, E. *coli*, and S. *agalactiae*, which were isolated from milk samples collected from she-camels with mastitis (Bacteriology Unit, King Faisal University). Whole blood (1 ml) was diluted with 0.9 ml medium (RPMI-1640, Sigma-Aldrich, Deisenhofen, Germany) in sterile 12 × 75 mm glass tubes (BD Biosciences, San Jose, California, USA). Live bacterial suspension (0.1 ml; 10^7^ bacteria/ml) was added to the diluted blood and the mixture was then incubated for 6 h at 37 C. A negative control tube containing 1 ml blood and 1 ml medium without bacteria was also included. After incubation, the tubes were then put into icy water (to enhance the detachment of stimulated adherent cells from plastic) and immediately centrifuged at 4 °C for 10 min at 1000xg. After removing the supernatant, the cell pellet was suspended in PBS.

### Separation of blood leukocytes

Separation of whole camel leukocytes was done after hypotonic lysis of blood erythrocytes [28]. For leukocyte separation, 5 ml camel blood was diluted with PBS (1 : 2) and centrifuged at 1000 ×g for 10 min (4 °C) without break. After carefully removal of blood plasma, the erythrocytes were lysed by adding 5 ml distilled water for 20 s and subsequent addition of 5 ml double concentrated PBS to restore tonicity. After centrifugation at 500 ×g for 10 min (4 °C) with break, the cell pellet was resuspended. The erythrolysis was repeated (usually twice) until complete removal of red blood cells. Subsequently, the cells were suspended in 10 ml PBS and washed two times (250 ×g and 100 ×g for 10 min each) and finally adjusted to 5 × 10^6^ cells/ml in MIF buffer (PBS containing bovine serum albumin (5 g/L) and NaN3 (0.1 g/L)).

### Analysis of cell viability

**Fig. 1:**
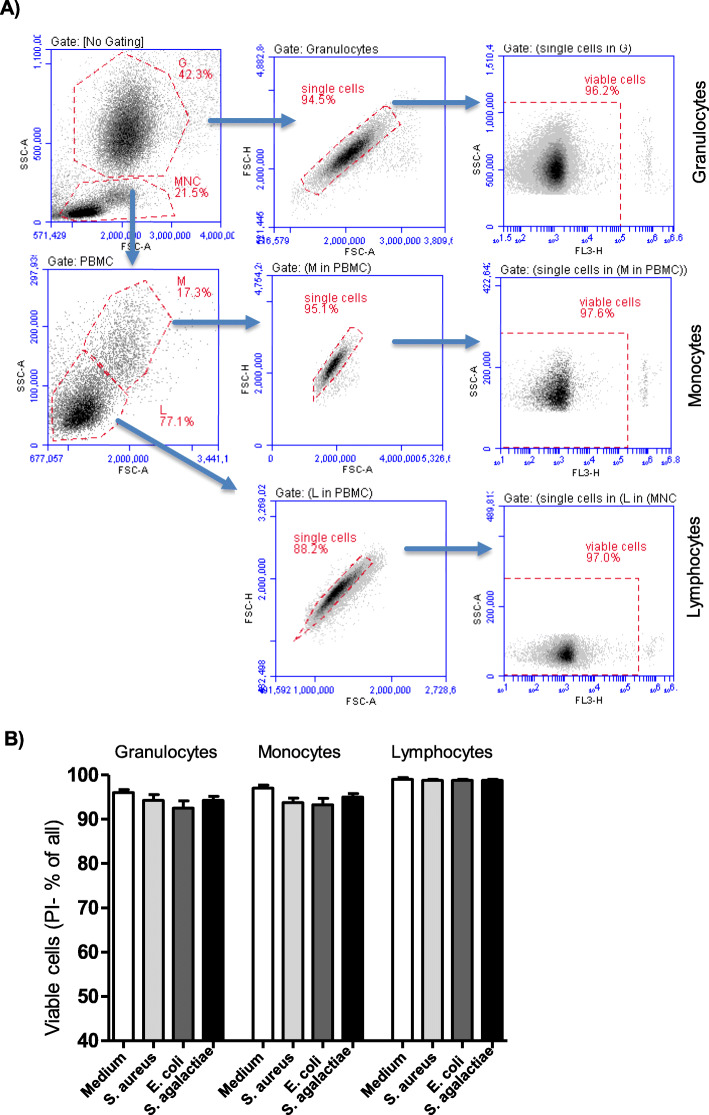
Gating strategy for the identification of the main leukocyte populations in camel blood and the analysis of their viability. (**A**) Separated camel leukocytes were labeled with propidium iodide and analyzed by flow cytometry. In a SSC/FSC dot plot, camel granulocytes (G) and mononuclear cells (MNC) were gated according to their forward and side scatter characteristics. In the MNC gate, monocytes (M) and lymphocytes (L) were identified based on their different forward and side scatter characteristics. In a separate FSC-A/FSC-H dot plot for each cell population, a gate was made on single cells (exclusion of duplets). In a SSC-A/FL-3 dot plot, viable cells were identified as PI-negative cells. (**B**) For unstimulated cells, cells stimulated with E. *coli*, S. *aureus*, and S. *agalactiae*, the percentages of viable cells among granulocytes, monocytes, and lymphocytes were calculated and data were presented as mean ± SEM

Cell viability of blood leukocytes was measured using the dye exclusion assay [29]. Stimulated and unstimulated leukocytes were incubated with the DNA-binding dye propidium iodide (PI; 2 µg/ml, Calbiochem, Germany) and were analyzed by flow cytometry (Fig. 1 A-B). PI uptake versus exclusion was used to discriminate dead cells with permeable plasma membranes (PI-positive) from live cells with intact membranes (PI-negative).

**Fig. 2:**
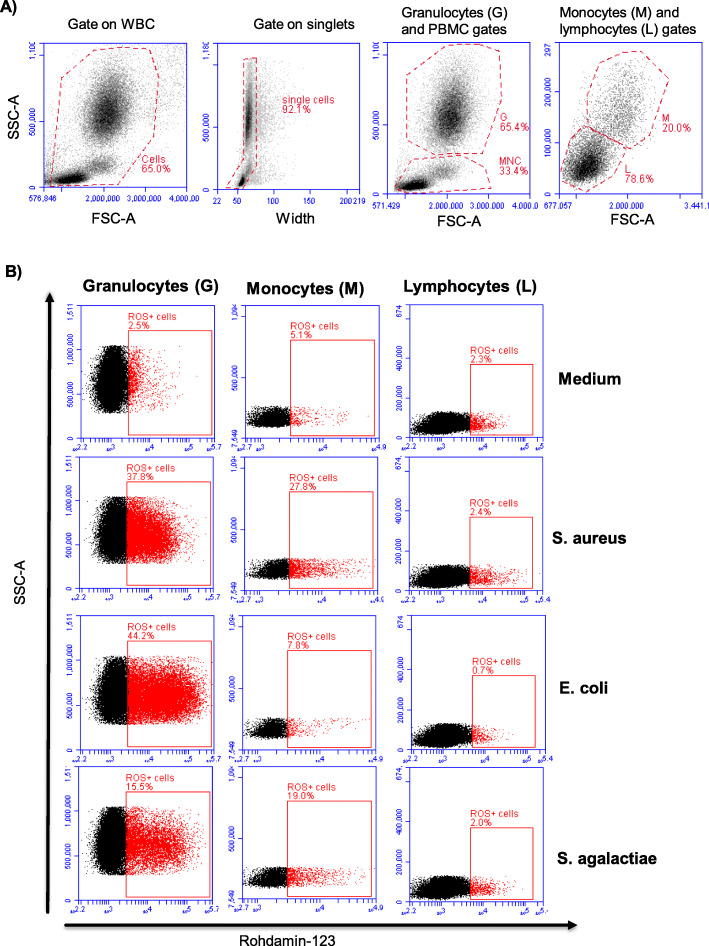
Flow cytometric analysis of ROS production in camel blood leukocytes. Camel leukocytes were separated from unstimulated and stimulated blood and separated cells were labeled with the ROS-sensitive dye DHR-123 and labeled cells were analyzed by flow cytometry A) In a SSC-A/FSC-A dot plot, a gate was seton leukocytes and duplets were excluded in a FSC-A/FSC-H dot plot. Camel granulocytes (G) and mononuclear cells (MNC) were gated according to their forward and side scatter characteristics. In the MNC gate, monocytes (M) and lymphocytes (L) were identified based on their different forward and side scatter characteristics. In a SSC-A/FL-1 dot plot, the percentage of DHR-positive cells and the mean green fluorescence intensity for each cell population were calculated

### Monoclonal antibodies

Monoclonal antibodies used in this study are listed in Table 1.

**Table 1:** List of antibodies

Antigen	Antibody clone	Label	Source	Isotype
CD14	TÜK4	-	WSU	mIgG1
MHCII	TH81A5	-	Kingfisher	mIgG2a
CD11a	G43-25B	PE	BD	mIgG2a
CD18	6.7	FITC	BD	mIgG1
mIgG2a	polyclonal	PE	Invitrogen	gIgG
mIgG1	polyclonal	FITC	Invitrogen	gIgG

*Ig* Immunoglobulin, *m* mouse, *MHCII* Major Histocompatibility Complex class II, *FITC* Fluorescein isothiocyanate, *PE* Phycoerythrin, *PerCP* Peridinin-Chlorophyll-Protein, *BD* Becton Dickinson

### Membrane immunofluorescence and flow cytometry

The expression of different myeloid markers and cell adhesion molecules was analyzed using membrane immunofluorescence and flow cytometry [30]. Separated leukocytes (4 × 10^5^) were incubated with unlabeled primary monoclonal antibodies (mAbs) specific for the cell markers CD14 and MHCII or with directly labeled monoclonal antibodies to the cell adhesion molecules CD11a and CD18 [31]. After incubation (15 min; 4 °C), cells were washed twice and cells labeled with anti-CD14 and anti-MHC class II molecules were incubated with mouse secondary antibodies (IgG1, IgG2a; Invitrogen) labeled with different fluorochromes. Mouse isotype control antibodies (Becton Dickinson Biosciences) were also included. Washed cells were analyzed using the Accurie C6 flow cytometer (BD Biosciences). At least 100 000 total leukocytes were collected and analyzed with the CFlow Software, Version 1.0.264.21 (BD Biosciences).

### Generation of reactive oxygen species (ROS)

ROS generation was performed in 96-well round-bottom microtiter plates (Corning, NY, USA) [32]. Stimulated and non-stimulated leukocytes (1 × 10^6^/well) were incubated for 20 min (37 °C, 5 % CO_2_) with the ROS-sensitive dye dihydrorhodamine (DHR)-123, (750 ng/ml final, Mobitec, Goettingen, Germany). After incubation, cells were washed with MIF buffer, and the percentage of ROS-positive cells and the relative amount of generated ROS was determined by flow cytometry (Accurie C6 flow cytometer, BD Biosciences) after acquisition of 100 000 events (Fig. 2A-B).

### Statistical analyses

Statistical analysis was carried out using the software Prism (GraphPad software version 5). Results are expressed as mean ± S.E. of the mean (SEM). Differences between means were tested with one-factorial analysis of variance (ANOVA). Results were considered statistically significant at a p-value of less than 0.05.

## Results

### Leukocyte viability is not affected by in-vitro stimulation with live bacteria

The mean percentage of viable cells (PI-negative) among unstimulated cells (cells in medium control) was 99 % ± 0.3 for lymphocytes, 97 % ± 0.6 for monocytes, and 96 % ± 0.6 for neutrophils. In comparison to stimulation with other bacteria, stimulation with E. *coli* induced the lowest percentage of viable cells among neutrophils (92 % ± 1.6) and monocytes (93 % ± 1.4). However for all leukocyte populations, no significant changes (p > 0.05) were found in the fraction of viable cells after bacterial stimulation (Fig. 1 A and B).

### Bacterial stimulation differently induces ROS-production in camel leukocyte populations

**Fig. 3:**
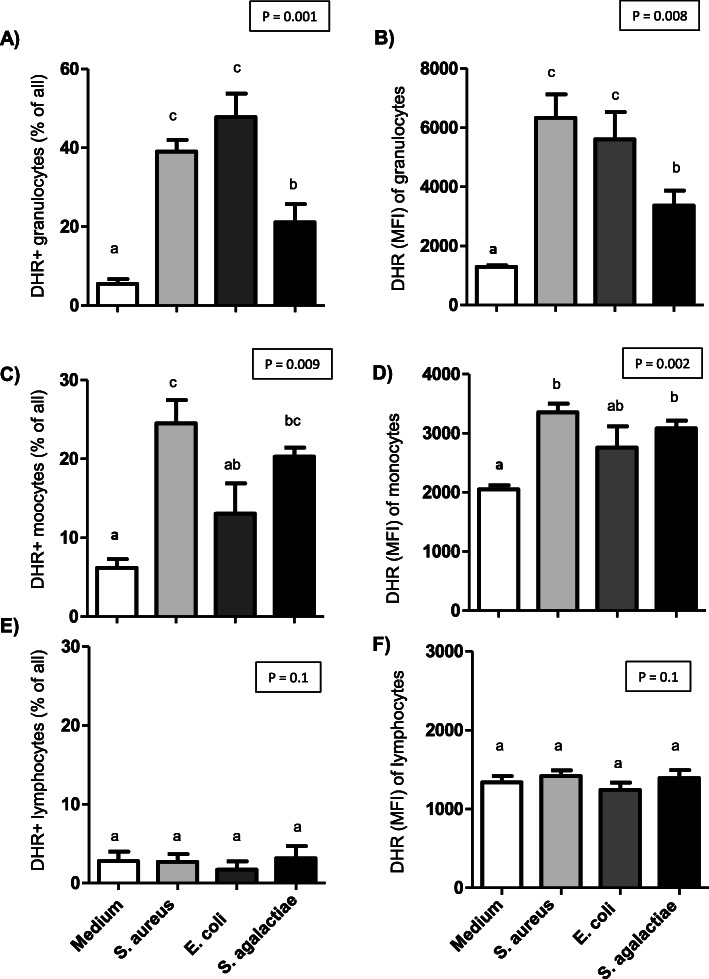
Effect of bacterial species on ROS production in camel blood leukocytes. For unstimulated cells, cells stimulated with E. *coli*, S. *aureus*, and S. *agalactiae*, the percentages of DHR-positive cells and the mean green fluorescence intensity for each cell population were calculated. Data for monocytes (M) and lymphocytes (L) was presented as mean ± SEM. Different lowercase superscript letters indicate statistical significance (*P* < 0.05.)

In comparison to unstimulated granulocytes, all bacterial species induced a significant (p < 0.05) rise in the percentage of ROS-positive granulocytes (Fig. 3 A) and the amount of ROS produced by granulocytes as measured by the mean fluorescence intensity (MFI) of the ROS-sensitive dye DHR-123 (Fig. 3B). The comparison between the three bacterial species revealed that stimulation with S. *aureus* (39.1 % ± 2.9) or *E. coli* (47.8 % ± 6.0) resulted in a significantly (p < 0.05) higher percentage of ROS-positive granulocytes in comparison to stimulation with *S. agalactiae* (21.1 % ± 4.1). In addition, the increase in the amount of ROS produced by granulocytes (fold increase relative to medium control) was significantly higher after stimulation with *S. aureus* (4.9 fold ± 0.6) or *E. coli* (4.3 fold ± 0.6) than *S. agalactiae* (2.6 ± 0.4). In monocytes, only stimulation with *S. aureus* or *S. agalactiae* induced a significant (p < 0.05) increase in the percentage of ROS-positive cells (Fig. 3 C) and the amount of ROS produced by monocytes in comparison to unstimulated cells (Fig. 3D). In contrast to this, stimulation with *E. coli* did not significantly change the percentage of ROS-positive monocytes or the amount of ROS produced by monocytes (p > 0.05). For lymphocytes, none of the bacterial species induced significant changes (p > 0.05) in the percentage of ROS-positive cells or the MFI of DHR-123 (Fig. 3E, F).

### Impact of bacterial stimulation on the expression of cell adhesion molecules on camel leukocytes

**Fig. 4:**
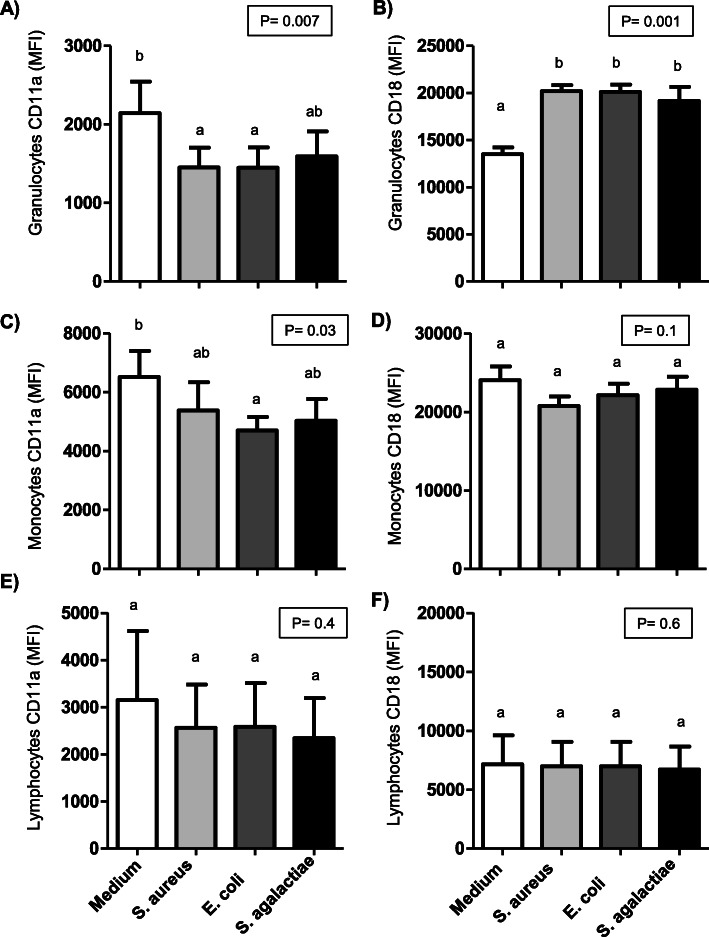
Adhesion molecules expression on leukocyte populations in camel blood. Camel leukocytes were separated from unstimulated and stimulated blood and separated cells were labeled with monoclonal antibodies to CD11a and CD18 and labeled cells were analyzed by flow cytometry. For granulocytes (**A**-**B**), monocytes (**C**-**D**), and lymphocytes (**E**-**F**), the expression density (mean fluorescence intensity; MFI) of CD11a and CD18 was calculated. Data for unstimulated and stimulated cells was presented as mean ± SEM. Differences between groups were considered significant if *P* < 0.05

While the expression of the cell adhesion molecules CD11a and CD18 on blood lymphocytes was not affected significantly (*p* > 0.05) by bacterial stimulation (Fig. 4E, F), the expression level of the two molecules on neutrophils and monocytes was differently affected by bacterial stimulation. CD11a expression on granulocytes was significantly (*p* < 0.05) reduced after stimulation with *S. aureus* or *E. coli* (Fig. 4 A), whereas only *E. coli* induced the reduction (*p* < 0.05) of CD11a expression on monocytes (Fig. 4 C). Only for granulocytes, all bacterial species induced an increased (*p* < 0.05) expression of CD18 after stimulation (Fig. 4B).

### Impact of bacterial stimulation on the composition of blood monocytes

**Fig. 5:**
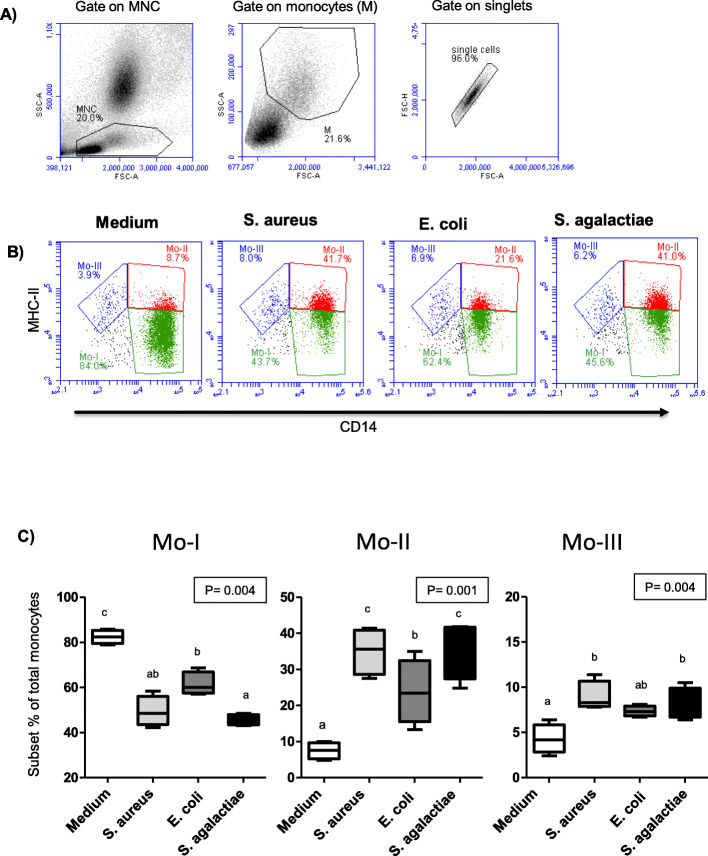
Impact of bacterial stimulation on the composition of camel monocytes. Camel leukocytes were separated from unstimulated and stimulated blood and were labeled with monoclonal antibodies to CD14 and MHCII molecules. Labeled cells were analyzed by flow cytometry. (**A**) Gating strategy of camel monocyte subsets. After setting agate on MNC, Camel monocytes were identified based on their forward (FSC) and side scatter (SSC) properties. In a separate FSC-A/FSC-H dot plot, a gate was made on single cells (exclusion of duplets). (**B**) Monocyte subsets Mo-I, Mo-II, and Mo-III were identified according to their CD14 and MHCII expression density (Mo-I: CD14^high^MHCII^low^, Mo-II: CD14^high^MHCII^high^, Mo-III: CD14^low^MHCII^high^). (**C**) The percentage of each monocyte subset was calculated and presented for unstimulated and stimulated blood. Differences between groups were considered significant (*) if *P* < 0.05

In unstimulated blood, the monocyte population consisted of the CD14^high^MHCII^low^ major monocyte subset (Mo-I; 82.4 % ± 1.5 of total monocytes), the CD14^high^MHCII^high^ inflammatory monocytes (Mo-II; 7.5 % ± 1.2 of total monocytes) and the CD14^low^MHCII^high^ monocyte subset (Mo-III; 4.3 % ± 0.8 of total monocytes) (Fig. 5B and C). Stimulation with either of the three bacterial species induced a significant (*p* < 0.05) decrease in fraction of Mo-I (49.4 % ± 3.4 for *S. aureus*, 61.5 % ± 26 for *E. coli* and 45.7 % ± 1.2 for *S. agalactiae*) with a significant 3 to 5 fold expansion (*p* < 0.05) of inflammatory Mo-II (4.8 fold ± 0.3 for *S. aureus*, 3.3 fold ± 0.6 for *E. coli* and 5.0 fold ± 0.8 for *S. agalactiae*) in comparison to unstimulated control (Fig. 5B and C). In addition, the fraction of Mo-III also increased (*p* < 0.05) after stimulation with *S. aureus* (9.0 ± 0.8 % of total monocytes) and *S. agalactiae* (8.2 ± 0.8 % of total monocytes) in comparison to unstimulated control (Fig. 5B and C).

### Bacterial stimulation enhances MHC-II expression on blood granulocytes

**Fig. 6:**
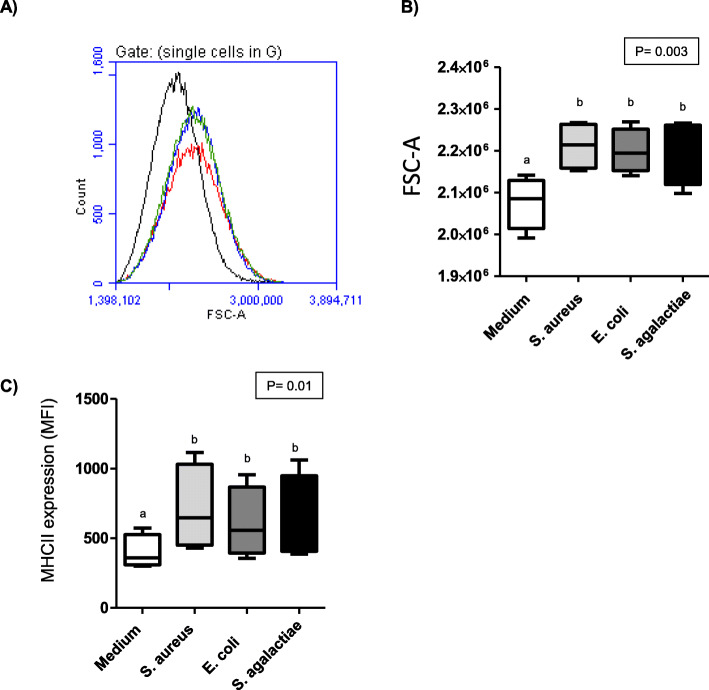
Stimulation-induced shape change and change in MHC-II expression in camel granulocytes. (**A**) Shape change in granulocytes was measured as mean fluorescence intensity (MFI) of FSC-A of gated granulocytes. (**B**) Data for unstimulated cells and cells stimulated with different bacterial species were calculated and presented as means ± SEM (* = *p* < 0.05). (**C**) MHC-II expression density on granulocytes from unstimulated blood and blood stimulated with different bacterial species was calculated and presented as means ± SEM (* = *p* < 0.05)

Bacterial stimulation of whole blood activated significantly (*p* < 0.05) blood granulocytes as measured by increased forward scatter (FSC) values (Fig. 6 A and B). The stimulation-induced increase in granulocytes FSC was comparable between all bacterial species (*p* > 0.05). In addition, stimulation with either of the bacterial species elicited the upregulation (*p* < 0.05) of cell surface MHC-II molecules on granulocytes (Fig. 6 C). The increase was, however, highest on cells stimulated with *S. aureus* (MFI 710 ± 154) in comparison to unstimulated blood (MFI 399 ± 61).

## Discussion

Pathogen-species-specific effects on several components of the innate and adaptive immune responses have been described for several species [8, 9]. *Escherichia coli (E. coli)*, *Staphylococcus aureus* (*S*. *aureus*), and *Streptococcus agalactiae* (*S*. *agalactiae*) are responsible for several infectious diseases in the dromedary camel [2, 10]. The current study investigated the modulatory effects of whole blood stimulation with the bacterial species, *E. coli*, *S. aureus*, or *S. agalactiae*, on the phenotype and function of camel leukocytes. The whole-blood culture used in the current study has been widely proven as an effective stimulation method, preserving the microenvironment of interaction between the pathogen and immune cells as it presents in vivo [33, 34].

Monocytes and neutrophils are innate immune cells with key roles during the immune response to bacterial infections [35].In a recent report, three heterogenic subpopulations of monocytes have been described in dromedary camels based on the expression profiles of major histocompatibility complex (MHC) class II molecules and CD14 [24]. Subset one (Mo-I) expresses high levels of CD14 and low levels of MHC class II and is the most abundant monocyte subset in blood. Subset two (Mo-II) is a minor subset of monocytes, which expresses high levels of CD14 and MHC class II and is considered the inflammatory monocytes with increased phagocytic and reactive oxygen species (ROS) production activity. While subset three (Mo-III) is another minor subpopulation of monocytes with low levels of CD14 and high levels of MHC class II [24]. In the current study, stimulation with either of the bacterial species significantly reduced the fraction of Mo-I and induced an expansion in the inflammatory Mo-II. This effect seems in line with the reported expansion in camel Mo-II in she-camels with *E. coli- or S. aureus-*caused clinical endometritis [22]. In human, an expansion of the intermediate CD14^high^ MHC-II^high^ monocytes was observed in several bacterial infectious diseases [36, 37]. This indicates functional similarity between camel Mo-II and human intermediate monocytes. For camel Mo-III, only stimulation with *S*. *aureus* or *S*. *agalactiae* but not with *E. coli* elicited an increase in the number of M-III monocytes. Whether this effect is unique to gram-positive bacteria, still to be investigated.

According to recent reports, granulocytes can acquire the function of antigen-presenting cells and contribute to adaptive immune responses by activating T cells in an MHC class II-dependent manner [26]. The stimulation-induced upregulation of MHC class II molecules on camel blood granulocytes although supports the reported role of granulocytes as antigen-presenting cells, seems, however, in contrast to the human system, where the upregulation of MHC class II molecules on granulocytes required antigen-specific stimuli, whereas exposure to innate Toll-like receptor ligands was not sufficient [25].

The generation of ROS is a key antimicrobial function of phagocytes that serves the effective killing of bacteria [38]. In the current study, *E. coli*, in contrast to *S. aureus* and *S. agalactiae*, failed to stimulate ROS production in camel monocytes. This seems different from cattle, where stimulation with *E. coli* elicited ROS generation in blood monocytes [27]. It is also contrary to the pro-inflammatory nature of *E.coli*-induced infections [13]. For granulocytes, although stimulation with any of the three pathogen species elicited ROS production, the magnitude of the response was significantly lower toward stimulation with *S. agalactiae* compared to stimulation with *S. aureus* or *E. coli.* This is also the case for the stimulation-induced change in CD11a expression on granulocytes, being only induced after stimulation with *S. aureus* or *E. coli*. The biological significance of the change in adhesion molecules expression on stimulated cells and its impact on cell migration and the innate immune response needs to be analyzed in functional studies.

Several reports have shown that the differences in the immune response to different bacterial species may correspond to the variation in the cell surface receptors stimulated by different bacterial pathogen-associated molecular patterns (PAMPs) [39]. According to studies in other species, the pattern recognition receptor Toll-like receptor 4 (TLR-4) interact with gram-negative bacteria through the recognition of the cell wall component lipopolysaccharide (LPS) in complex with the LPS-binding protein and the monocytic marker CD14, while TLR-2 interacts with lipoteichoic acids and peptidoglycan in gram-positive bacteria [39–41]. The characterization of the expression pattern of PRRs, including TLR in camel myeloid cells is essential for obtaining a deeper understanding of the underlying mechanisms of the heterogeneity in the responevness of camel monocytes and neutrophils toward the studied bacterial species. The heterogeneity in the response of camel phagocytes to stimulation with *S. aureus* and *S. agalactiae*, two gram-positive bacteria, indicates the existence of further mechanisms for the pathogen-species specific modulatory effects on the immune system.

## Conclusions

The different responsiveness of monocytes and granulocytes toward different bacterial species indicates different host-pathogen interaction mechanisms for the two innate cell populations in the dromedary camel. Whether this is due to different sets of pattern recognition receptors on camel monocytes and granulocytes, still to be investigated. In addition, the phenotypic and functional differences between cells stimulated with *E. coli*, *S. aureus*, or *S. agalactiae* argues for pathogen-species-specific effects of the bacterial pathogens on the camel innate myeloid cells, monocytes and neutrophils. Further work is needed to understand the mechanisms involved in the heterogenic response of innate immune cells toward different bacterial species.

## Data Availability

The datasets used and/or analyzed during the current study are available on reasonable request.

## Abbreviations

*E.coli*: *Escherichia coli*
*S*.* aureus*: *Staphylococcus aureus*
*S*. *agalactiae*: *Streptococcus agalactiae*
ROS: Reactive oxygen species
MHC: Major histocompatibility complex
CD: Cluster of differentiation
MFI: Mean fluorescence intensity
Mo-I: Camel monocyte subset I
Mo-II: Camel monocyte subset II
Mo-III: Camel monocyte subset III
MCP: Monocyte chemotactic protein
MIP: Macrophage inflammatory protein
TLR: Toll-like receptor
PAMP: Pathogen-associated molecular pattern
PRR: Pattern recognition receptor

## References

[1] Bannerman DD, Paape MJ, Hare WR, Hope JC (2004). Characterization of the bovine innate immune response to intramammary infection with Klebsiella pneumoniae. J Dairy Sci.

[2] El Tigani-Asil ETA, Abdelwahab GE, Veedu J, Khalafalla AI, Mohamed ZSA, Ishag HZA, Shah AAM, Alhosani MAA, Al Muhairi SSM (2020). Gangrenous mastitis in dromedary camels in UAE caused by Streptococcus agalactiae. BMC Vet Res.

[3] Gunther J, Koy M, Berthold A, Schuberth HJ, Seyfert HM (2016). Comparison of the pathogen species-specific immune response in udder derived cell types and their models. Vet Res.

[4] Gunther J, Petzl W, Bauer I, Ponsuksili S, Zerbe H, Schuberth HJ, Brunner RM, Seyfert HM (2017). Differentiating Staphylococcus aureus from Escherichia coli mastitis: S. aureus triggers unbalanced immune-dampening and host cell invasion immediately after udder infection. Sci Rep.

[5] Keefe GP (1997). Streptococcus agalactiae mastitis: a review. Can Vet J.

[6] Landwehr-Kenzel S, Henneke P (2014). Interaction of Streptococcus agalactiae and Cellular Innate Immunity in Colonization and Disease. Front Immunol.

[7] Petzl W, Zerbe H, Gunther J, Seyfert HM, Hussen J, Schuberth HJ (2018). Pathogen-specific responses in the bovine udder. Models and immunoprophylactic concepts. Res Vet Sci.

[8] Heimes A, Brodhagen J, Weikard R, Seyfert HM, Becker D, Meyerholz MM, Petzl W, Zerbe H, Hoedemaker M, Rohmeier L (2020). Hepatic Transcriptome Analysis Identifies Divergent Pathogen-Specific Targeting-Strategies to Modulate the Innate Immune System in Response to Intramammary Infection. Front Immunol.

[9] Sela U, Euler CW, Correa da Rosa J, Fischetti VA (2018). Strains of bacterial species induce a greatly varied acute adaptive immune response: The contribution of the accessory genome. PLoS Pathog.

[10] Tibary A, Fite C, Anouassi A, Sghiri A (2006). Infectious causes of reproductive loss in camelids. Theriogenology.

[11] Abera M, Abdi O, Abunna F, Megersa B (2010). Udder health problems and major bacterial causes of camel mastitis in Jijiga, Eastern Ethiopia: implication for impacting food security. Trop Anim Health Prod.

[12] Bekele T, Molla B (2001). Mastitis in lactating camels (Camelus dromedarius) in Afar Region, north-eastern Ethiopia. Berl Munch Tierarztl Wochenschr.

[13] Burvenich C, Van Merris V, Mehrzad J, Diez-Fraile A, Duchateau L (2003). Severity of E. coli mastitis is mainly determined by cow factors. Vet Res.

[14] Keefe G (2012). Update on control of Staphylococcus aureus and Streptococcus agalactiae for management of mastitis. The Veterinary clinics of North America Food animal practice.

[15] Keane OM. Symposium review: Intramammary infections-Major pathogens and strain-associated complexity. J Dairy Sci. 2019;102(5):4713–4726. 10.3168/jds.2018-15326 30827546

[16] Wolf C, Kusch H, Monecke S, Albrecht D, Holtfreter S, von Eiff C, Petzl W, Rainard P, Broker BM, Engelmann S (2011). Genomic and proteomic characterization of Staphylococcus aureus mastitis isolates of bovine origin. Proteomics.

[17] Duan J, Xie Y, Yang J, Luo Y, Guo Y, Wang C (2016). Variation of Circulating Inflammatory Mediators in Staphylococcus aureus and Escherichia coli Bloodstream Infection. Med Sci Monit.

[18] Ng LG, Ostuni R, Hidalgo A (2019). Heterogeneity of neutrophils. Nat Rev Immunol.

[19] Yang PQ, Li YH, Xie Y, Liu Y. Different Faces for Different Places: Heterogeneity of Neutrophil Phenotype and Function. J Immunol Res. 2019;2019. 10.1155/2019/8016254 PMC6421822 30944838

[20] Xiong HZ, Pamer EG (2015). Monocytes and infection: Modulator, messenger and effector. Immunobiology.

[21] Ali A, Tharwat M, Al-Sobayil FA (2010). Hormonal, biochemical, and hematological profiles in female camels (Camelus dromedarius) affected with reproductive disorders. Anim Reprod Sci.

[22] Hussen J, Shawaf T, Al-Mubarak AIA, Al Humam NA, Almathen F, Schuberth HJ. Leukocyte populations in peripheral blood of dromedary camels with clinical endometritis. Anim Reprod Sci. 2020. 10.1016/j.anireprosci.2020.106602 32980651

[23] Hussen J, Shawaf T, Schuberth MJ. HJ: Whole blood stimulation with lipopolysaccharide modulates phenotype and function of dromedary camel neutrophils. Journal of Camel Practice Research. 2019;26(1):105–10.

[24] Hussen J, Shawaf T, Al-Mubarak AIA, Al Humam NA, Almathen F, Schuberth HJ (2020). Dromedary camel CD14(high) MHCII(high) monocytes display inflammatory properties and are reduced in newborn camel calves. BMC Vet Res.

[25] Vono M, Lin A, Norrby-Teglund A, Koup RA, Liang F, Lore K (2017). Neutrophils acquire the capacity for antigen presentation to memory CD4(+) T cells in vitro and ex vivo. Blood.

[26] Lin A, Lore K (2017). Granulocytes: New Members of the Antigen-Presenting Cell Family. Front Immunol.

[27] Hussen J, Duvel A, Sandra O, Smith D, Sheldon IM, Zieger P, Schuberth HJ (2013). Phenotypic and functional heterogeneity of bovine blood monocytes. PLoS One.

[28] Hussen J, Shawaf T, Al-Herz AI, Alturaifi HR, Alluwaimi AM (2017). Reactivity of commercially available monoclonal antibodies to human CD antigens with peripheral blood leucocytes of dromedary camels (Camelus dromedarius). Open Vet J.

[29] Crowley LC, Scott AP, Marfell BJ, Boughaba JA, Chojnowski G, Waterhouse NJ. Measuring Cell Death by Propidium Iodide Uptake and Flow Cytometry. Cold Spring Harb Protoc. 2016;2016(7). 10.1101/pdb.prot087163 27371595

[30] Eger M, Hussen J, Drong C, Meyer U, von Soosten D, Frahm J, Daenicke S, Breves G, Schuberth HJ (2015). Impacts of parturition and body condition score on glucose uptake capacity of bovine monocyte subsets. Vet Immunol Immunopathol.

[31] Hussen J, Schuberth HJ (2017). Heterogeneity of Bovine Peripheral Blood Monocytes. Front Immunol.

[32] Hussen J, Koy M, Petzl W, Schuberth HJ (2016). Neutrophil degranulation differentially modulates phenotype and function of bovine monocyte subsets. Innate immunity.

[33] Damsgaard CT, Lauritzen L, Calder PC, Kjaer TM, Frokiaer H (2009). Whole-blood culture is a valid low-cost method to measure monocytic cytokines - a comparison of cytokine production in cultures of human whole-blood, mononuclear cells and monocytes. J Immunol Methods.

[34] Gomes NE, Brunialti MK, Mendes ME, Freudenberg M, Galanos C, Salomao R (2010). Lipopolysaccharide-induced expression of cell surface receptors and cell activation of neutrophils and monocytes in whole human blood. Braz J Med Biol Res.

[35] Soehnlein O, Lindbom L (2010). Phagocyte partnership during the onset and resolution of inflammation. Nat Rev Immunol.

[36] Ziegler-Heitbrock L (2014). Monocyte subsets in man and other species. Cell Immunol.

[37] Ziegler-Heitbrock L (2007). The CD14 + CD16 + blood monocytes: their role in infection and inflammation. J Leukoc Biol.

[38] Dupre-Crochet S, Erard M, Nubetae O (2013). ROS production in phagocytes: why, when, and where?. J Leukoc Biol.

[39] Rockel C, Hartung T (2012). Systematic review of membrane components of gram-positive bacteria responsible as pyrogens for inducing human monocyte/macrophage cytokine release. Front Pharmacol.

[40] Schmidt RR, Pedersen CM, Qiao Y, Zahringer U (2011). Chemical synthesis of bacterial lipoteichoic acids: an insight on its biological significance. Org Biomol Chem.

[41] Hadley JS, Wang JE, Foster SJ, Thiemermann C, Hinds CJ (2005). Peptidoglycan of Staphylococcus aureus upregulates monocyte expression of CD14, Toll-like receptor 2 (TLR2), and TLR4 in human blood: possible implications for priming of lipopolysaccharide signaling. Infect Immun.

